# ASO Author Reflections: The Challenge of Peritoneal Metastases in Patients with Small Bowel Neuroendocrine Tumors

**DOI:** 10.1245/s10434-025-18587-w

**Published:** 2025-11-05

**Authors:** Jeremy Chang, Luis C. Borbon, Scott K. Sherman, Po Hien Ear, Andrew M. Bellizzi, Joseph S. Dillon, James R. Howe

**Affiliations:** 1 Department of Surgery, University of Iowa Carver College of Medicine, Iowa City, IA USA; 2 Department of Pathology, University of Iowa Carver College of Medicine, Iowa City, IA USA; 3 Department of Internal Medicine, University of Iowa Carver College of Medicine, Iowa City, IA USA

## Past

Peritoneal carcinomatosis is a vexing problem in gastrointestinal (GI) cancers and can lead to many complications, including pain, bowel obstruction, and death. Peritoneal metastases (PM) are a common site of disease spread for small bowel neuroendocrine tumors (SBNETs). The biology of NETs, however, is different from that of other GI tumors, often characterized by slow growth. Current management for PM in SBNET involves a combination of surgical resection and medical systemic therapies [i.e., somatostatin analogs, chemotherapy and biologics, and peptide receptor radionuclide therapy (PRRT)].^1^ The North American Neuroendocrine Tumor Society recommends aggressive cytoreduction of peritoneal disease, however, these treatment decisions can be nuanced and should be personalized to patients and their distribution of disease.^2^ Studies providing long-term outcomes using this approach have been limited.

## Present

This study finds that cytoreduction of PM in patients with SBNET leads to favorable oncologic outcomes with a median overall survival of 109 months.^3^ This supports previous studies that collectively have found that a comprehensive cytoreduction of PM [to a closing Lyon score of < 1 or a completeness of cytoreduction (CC) score of < 1] and liver metastases (removing > 70% of liver disease) is associated with improved outcomes.^4^^,^^5^ We find, however, that these cytoreductive interventions are associated with higher rates of major complications. PRRT, while increasingly used as a management option, has a higher rate of associated small bowel obstruction than previously reported, especially in patients with SBNET with PM. Continued surveillance of high-risk patients (i.e., those with high T stage or liver metastases) is important, as 5% of those without PM on presentation will develop PM at a median of 54 months.

## Future

These results suggest that surgical intervention improves survival in patients with SBNET with PM when cytoreduction is performed. However, appropriate patient selection is vital in achieving these outcomes. Routine preoperative functional somatostatin receptor (SSTR) imaging is critical to understanding disease burden, location, and operative planning. While PRRT is an option, adverse effects such as small bowel obstruction are more common than previously reported in clinical trials. We recommend surgical intervention as a first-line treatment of PM in patients with SBNET. Because of the complexity and rarity of this disease, management of PM in patients with SBNET is best performed through a multidisciplinary approach.
